# The associations among quantitative spectral CT parameters, Ki-67 expression levels and EGFR mutation status in NSCLC

**DOI:** 10.1038/s41598-020-60445-0

**Published:** 2020-02-26

**Authors:** Liaoyi Lin, Jiejun Cheng, Daoqiang Tang, Ying Zhang, Feng Zhang, Jianrong Xu, Handong Jiang, Huawei Wu

**Affiliations:** 1 Department of Radiology, Renji Hospital, School of Medicine, Shanghai Jiao Tong University, No.160, Pujian Road, Shanghai, 200127 China; 2 Department of Pathology, Renji Hospital, School of Medicine, Shanghai Jiao Tong University, No.160, Pujian Road, Shanghai, 200127 China; 3 Department of Respiratory, Renji Hospital, School of Medicine, Shanghai Jiao Tong University, No.160, Pujian Road, Shanghai, 200127 China

**Keywords:** X-ray tomography, Cancer imaging

## Abstract

Dual-energy spectral computed tomography (DESCT) is based on fast switching between high and low voltages from view to view to obtain dual-energy imaging data, and it can generate monochromatic image sets, iodine-based material decomposition images and spectral CT curves. Quantitative spectral CT parameters may be valuable for reflecting Ki-67 expression and EGFR mutation status in non-small-cell lung cancer (NSCLC). We investigated the associations among the quantitative parameters generated in DESCT and Ki-67 expression and EGFR mutation in NSCLC. We studied sixty-five NSCLC patients with preoperative DESCT scans, and their specimens underwent Ki-67 and EGFR evaluations. Statistical analyses were performed to identify the spectral CT parameters for the diagnosis of Ki-67 expression and EGFR mutation status. We found that tumour grade and the slope of the spectral CT curve in the venous phase were the independent factors influencing the Ki-67 expression level, and the area under the curve (AUC) of the slope of the spectral CT curve in the venous phase in the receiver operating characteristic analysis for distinguishing different Ki-67 expression levels was 0.901. Smoking status and the normalized iodine concentration in the venous phase were independent factors influencing EGFR mutation, and the AUC of the two-factor combination for predicting the presence of EGFR mutation was 0.807. These results show that spectral CT parameters may be useful for predicting Ki-67 expression and the presence of EGFR mutation in NSCLC.

## Introduction

Lung cancer is one of the most common causes of cancer death worldwide^1^, and non-small-cell lung cancer (NSCLC) is the most common pathological type. Many NSCLC patients suffer from recurrence after treatment, and treatment outcomes varied among patients with advanced disease. Currently, several predictive factors, such as histological subtypes or biomarkers, including Ki-67 and epidermal growth factor receptor (EGFR), have shown important clinical value in the treatment and prognosis of NSCLC patients.

Ki-67 is a nuclear protein that is expressed during all active phases of the cell cycle but is absent in G0. Thus, it is regarded as a cellular proliferation marker^2,3^. It has predictive value for the clinical course of various cancers, e.g., invasiveness, treatment response, recurrence and survival^4–7^. Regarding NSCLC, Ki-67 has also been recognized as a common biological marker in the evaluation of lung cancer and has been shown to have great potential as an important prognostic factor^8–10^. Analysis of Ki-67 in resected NSCLC tissues suggests that patients with high Ki-67 values may have a more negative prognosis and a high risk of recurrence, and Ki-67 can also predict survival after treatment^8,11,12^. In clinical practice, patients with EGFR mutations can be selected for treatment with EGFR tyrosine kinase inhibitors (EGFR-TKIs) for their sensitivity to EGFR-TKIs, and this treatment results in long survival, enhanced quality of life and decreased treatment-related side effects^13–15^. Ki-67 analysis in NSCLC tissues is always generated from biopsy analysis prior to surgery, which is an invasive procedure, and the results may be uncertain because of the small sample size in an individual case or a nonrepresentative sample selection. The gold standard for EGFR mutation testing also relies on the detection of tumour tissues from the biopsy or surgery^16^. Thus, exploring noninvasive methods to assess Ki-67 expression levels and the presence of EGFR mutation in NSCLC would be beneficial for lung cancer patients.

Recently, dual-energy computed tomography (CT), which generates both monochromatic image sets and iodine-based material decomposition images, has become increasingly used in diagnosing cancer patients^17^. One of the dual-energy CT technologies is spectral CT, which is based on fast switching between high and low voltages from view to view to obtain dual-energy imaging data. It is useful for scanning soft tissues and has a higher contrast-to-noise ratio than conventional multi-slice CT. It enables the collection of the quantitative iodine concentration on iodine-based material decomposition images and the reflection of the structural difference in tumours via attenuation changes as a function of photon energy (spectral CT curve); furthermore, dual-energy CT serves as a useful method for distinguishing between benign and malignant pulmonary lesions^18,19^. The quantitative iodine concentration and spectral CT curves in dual-energy spectral CT may be valuable for reflecting the expression of Ki-67 and EGFR mutation status in NSCLC. However, to date, there have been no specific studies in this area. Thus, the goal of our study was to investigate the potential association between the quantitative parameters generated in dual-energy spectral CT and Ki-67 expression levels and the presence of EGFR mutation in NSCLC.

## Results

### Patient information

Sixty-five patients with NSCLC who underwent dual-energy spectral CT examination and subsequently underwent radical surgery were included in our study. According to the results of Ki-67 staining, patients were divided into the Ki-67 low-level expression group (Ki-67 ≤ 30%) and the high-level expression group (Ki-67 index>30%). There were 40 patients in the low-level group and 25 patients in the high-level group (Table 1). There were significant differences in pathological grade, tumour diameter, and EGFR mutation status between the Ki-67 low-level expression group and the high-level expression group, but there were no significant differences in sex, age, smoking status, histological type, or tumour stage. According to the EGFR gene mutation results, patients were divided into the EGFR wild-type group and the EGFR mutation group; there were 36 patients in the EGFR wild-type group and 29 patients in the EGFR mutation group (Table 2). In the EGFR mutation group, there were 15 patients with Exon19 Del, 3 patients with Exon20 Ins, 9 patients with Exon21 L858R, 1 patient with Exon21 L861Q and 1 patient with Exon18 G719X mutations. There was a significant difference in smoking status and Ki-67 expression levels between the EGFR wild-type group and the EGFR mutant group, while sex, age, pathological grade, histological type, tumour stage, and tumour diameter were not significantly different.

**Table 1:** Clinical data of different Ki-67 expression levels in NSCLC.

Characteristics		Low level	High level	P value
Sex	Male	23	20	0.105
	Female	17	5	
Patient age (year)		64.20 ± 10.73	64.44 ± 9.99	0.929
Smoking status	Smoker	8	6	0.703
	Non-smoker	32	19	
Pathological grade	I + II	29	5	<0.001
	III	11	20	
Histological type	Adenocarcinoma	32	16	0.153
	Non-adenocarcinoma	8	9	
Tumour stage	I + II	31	20	0.811
	III + IV	9	5	
Tumour diameter (mm)		20.32 ± 6.94	25.94 ± 10.24	0.021
EGFR gene status	EGFR wild-type	17	19	0.008
	EGFR mutation	23	6

**Table 2:** Clinical data of the groups of EGFR mutation status in NSCLC.

Characteristics		EGFR wild-type	EGFR mutation	P value
Sex	Male	25	18	0.532
	Female	11	11	
Patient age (year)		64.06 ± 10.55	64.58 ± 10.33	0.839
Smoking status	Smoker	12	2	0.023
	Non-smoker	24	27	
Pathological grade	I + II	16	18	0.157
	III	20	11	
Histological type	Adenocarcinoma	23	25	0.080
	Non-adenocarcinoma	13	4	
Tumour stage	I + II	29	22	0.647
	III + IV	7	7	
Tumour diameter (mm)		23.72 ± 10.06	20.95 ± 6.58	0.187
Ki-67 expression	Low level (≤30%)	17	23	0.008
	High level (>30%)	19	6	

### Dual-energy spectral CT findings with Ki-67 expression levels and EGFR mutation status

**Figure 1:**
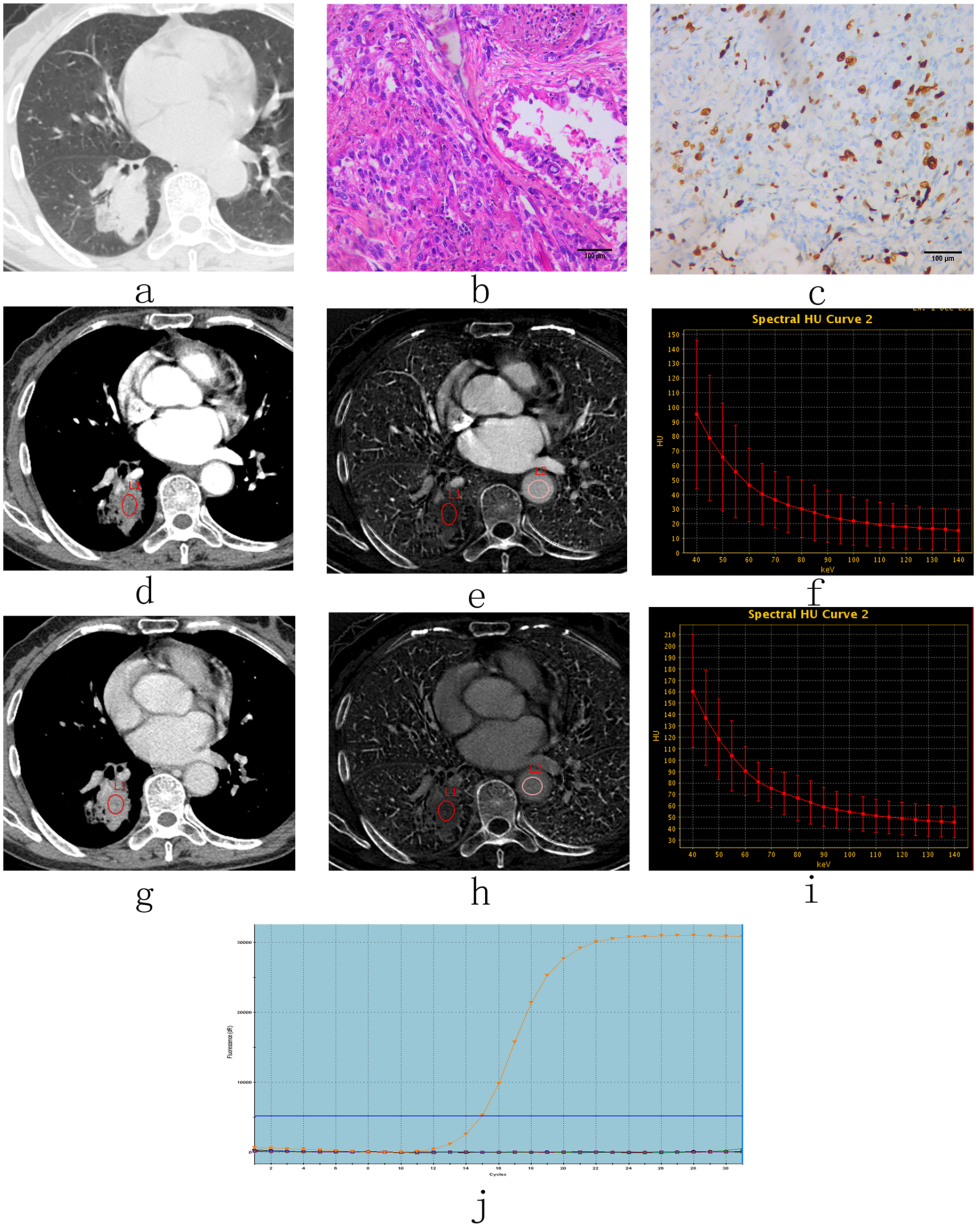
CT images and pathological analysis in a 65-year-old woman with high-level expression of Ki-67 in adenocarcinoma tissues and EGFR wild-type tissues. (**a**) Non-enhanced image with lung window settings. (**b**) Lung adenocarcinoma tissue with H&E staining (200x magnification). (**c**) Immunohistochemical staining for Ki-67 in lung adenocarcinoma tissues (200x magnification). There was high expression of Ki-67 in the nucleus. (**d–i**) 70 keV images after enhancement with mediastinal window settings, iodine-based images and spectral curves in AP (**d–f**) and VP (**g–i**). 70 keV CT value (AP)= 34.66 HU, 70 keV CT value (VP)=69.18 HU, NIC (AP) = 0.11, NIC (VP) = 0.3, λ_HU_ (AP) = 1.22, λ_HU_ (VP) = 1.76. (**j**) The molecular pathological results post-surgery reveal EGFR wild type (i.e., wild type). keV, kilo electron volt; NIC, normalized iodine concentration; λ_HU_, the slope of the spectral curve. AP, arterial phase; VP, venous phase.

**Figure 2:**
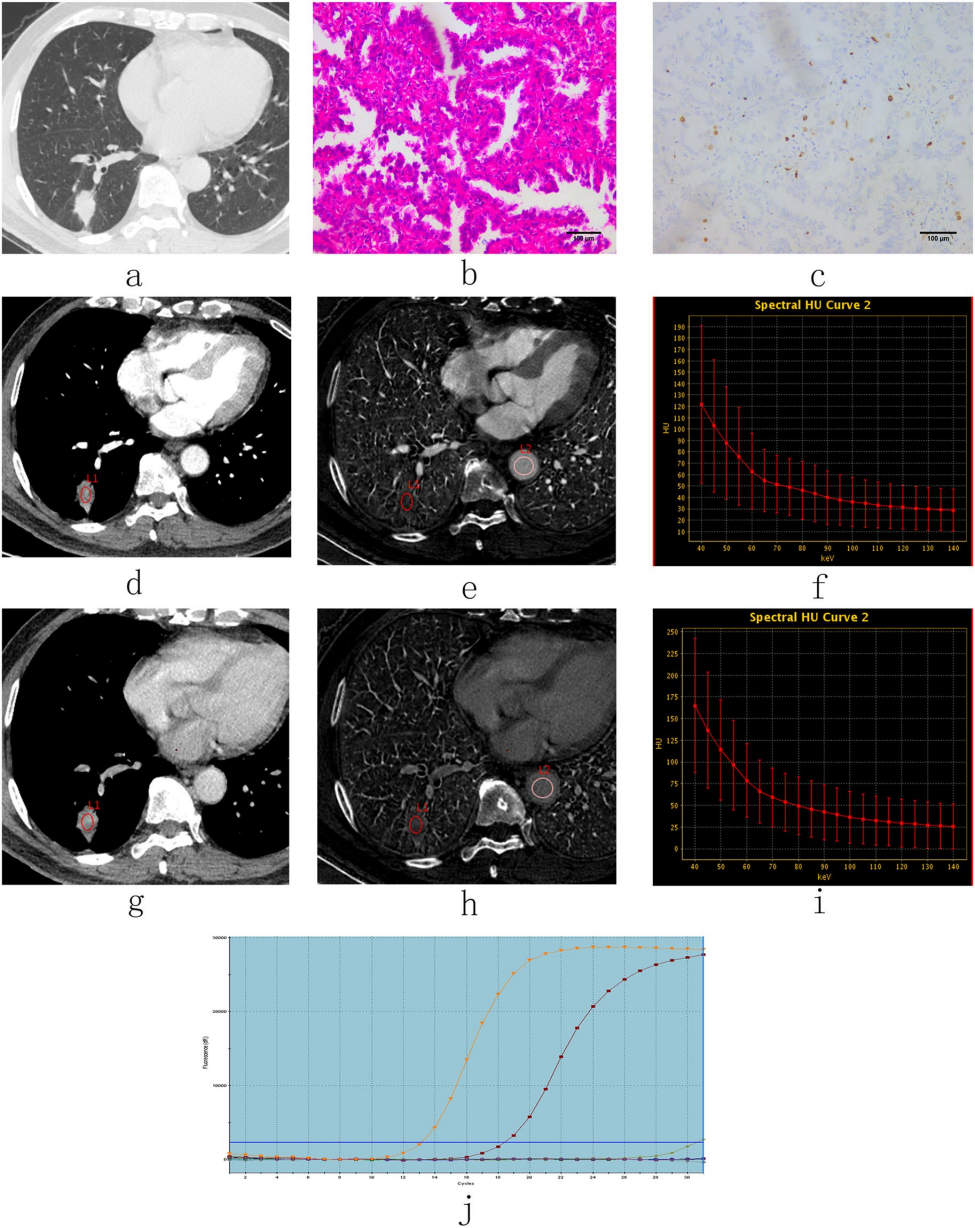
CT images and pathological analysis in a 69-year-old man with low-level expression of Ki-67 in adenocarcinoma tissues and Exon21 L858R EGFR mutation tissues. (**a**) Non-enhanced image with lung window settings. (**b**) Lung adenocarcinoma tissue with H&E staining (200x magnification). (**c**) Immunohistochemical staining for Ki-67 in lung adenocarcinoma tissues (200x magnification). There was low expression of Ki-67 in the nucleus. (**d–i**) 70 keV images after enhancement with mediastinal window settings, iodine-based images and spectral curves in AP (**d–f**) and VP (**g–i**). 70 keV CT value (AP)= 49.46 HU, 70 keV CT value (VP)= 75.40 HU, NIC (AP) = 0.15, NIC (VP) = 0.44, λ_HU_ (AP) = 1.43, λ_HU_ (VP) = 2.14. (**j**) The molecular pathological results post-surgery reveal the presence of EGFR mutations (i.e., Exon21 L858R). keV, kilo electron volt; NIC, normalized iodine concentration; λ_HU_, the slope of the spectral curve. AP, arterial phase; VP, venous phase.

The spectral CT quantitative parameters were different between the two different Ki-67 expression level groups and the two different EGFR gene statuses (Figs. 1 and 2).

The 70 keV CT values in the VP of the low-level Ki-67 expression tumours were significantly higher than those in the high-level Ki-67 expression tumours (64.63 ± 18.34 versus 52.40 ± 16.04, P = 0.008). The NIC values of the low-level Ki-67 expression tumours were significantly higher than those of the high-level Ki-67 expression tumours in both the AP (0.18 ± 0.08 versus 0.12 ± 0.04, P < 0.001) and VP (0.47 ± 0.11 versus 0.33 ± 0.07, P < 0.001). However, for the 70 keV CT values in the AP, there was no statistically significant difference between the low-level group and the high-level group (P > 0.05). The levels of λ_HU_ were also significantly higher in the low-level Ki-67 expression tumours than in the high-level Ki-67 expression tumours in both the AP (2.02 ± 0.93 versus 1.34 ± 0.42, P < 0.001) and VP (2.81 ± 0.87 versus 1.76 ± 0.32, P < 0.001) (Table 3).

**Table 3:** Comparison of spectral CT quantitative parameters between different Ki-67 expression levels in NSCLC.

Parameters	Low level	High level	P value
70 keV CT value in AP	45.83 ± 17.54	40.25 ± 16.71	0.209
70 keV CT value in VP	64.63 ± 18.34	52.40 ± 16.04	0.008
NIC in AP	0.18 ± 0.08	0.12 ± 0.04	<0.001
NIC in VP	0.47 ± 0.11	0.33 ± 0.07	<0.001
λ_HU_ in AP	2.02 ± 0.93	1.34 ± 0.42	<0.001
λ_HU_ in VP	2.81 ± 0.87	1.76 ± 0.32	<0.001

keV, kilo electron volt; NIC, normalized iodine concentration; λ_HU_, the slope of the spectral curve. AP, arterial phase; VP, venous phase.

The NIC values of the EGFR mutated tumours were significantly higher than those of the EGFR wild-type tumours in both the AP (0.18 ± 0.08 versus 0.13 ± 0.06, P = 0.032) and VP (0.47 ± 0.14 versus 0.37 ± 0.07, P < 0.001). The λ_HU_ values of the EGFR mutated tumours were significantly higher than those of the EGFR wild-type tumours in the VP (2.75 ± 1.06 versus 2.13 ± 0.56, P = 0.006), but the 70 keV CT values in the AP and VP and the λ_HU_ values in the AP demonstrated no statistically significant difference between the EGFR wild-type and the EGFR mutated tumours (P > 0.05) (Table 4).

**Table 4:** Comparison of spectral CT quantitative parameters between the EGFR nonmutated group and the EGFR mutant group in NSCLC.

Parameters	EGFR wild-type	EGFR mutation	P value
70 keV CT value in AP	43.65 ± 16.66	43.72 ± 18.39	0.987
70 keV CT value in VP	59.38 ± 18.28	60.61 ± 18.78	0.789
NIC in AP	0.13 ± 0.06	0.18 ± 0.08	0.032
NIC in VP	0.37 ± 0.07	0.47 ± 0.14	<0.001
λ_HU_ in AP	1.57 ± 0.60	1.98 ± 1.02	0.057
λ_HU_ in VP	2.13 ± 0.56	2.75 ± 1.06	0.006

keV, kilo electron volt; NIC, normalized iodine concentration; λ_HU_, the slope of the spectral curve. AP, arterial phase; VP, venous phase.

### The logistics regression analysis of the association of Ki-67 expression level with the spectral CT parameters and clinic characteristics

Logistic regression analysis was performed on the association between the Ki-67 expression level and the spectral CT parameters and clinical characteristics. In the univariate regression analysis, pathological grade, tumour diameter and EGFR mutation status as well as the spectral CT quantitative parameters of the 70 keV CT value in the VP, the NIC in the AP and VP, and the λ_HU_ value in the AP and VP were significantly correlated with Ki-67 expression levels.

Further multivariate regression analysis was used to correct for the interaction of various factors affecting Ki-67 expression. It was found that tumour pathological grade and λ_HU_ in the VP were independently correlated with Ki-67 expression. Between the Ki-67 high-level expression group and the Ki-67 low-level expression group, the odds ratio (OR) of tumour pathological grade (comparing grade III with grade I + II) for high-level Ki-67 expression was 5.291 with a 95% confidence interval of 1.166–24.010, and the OR of the λ_HU_ in the VP (increased 1 Unit) for high-level Ki-67 expression was 0.020 with a 95% confidence interval of 0.002–0.197 (Table 5).

**Table 5:** Univariate and multivariate logistic regression analyses of Ki-67 expression level.

	Univariate analysis	Multivariate analysis	
	P value	P value	OR (95% CI)
Sex (male versus female)	0.062		
Patient age	0.927		
Smoking status (non-smoker versus smoker)	0.703		
Pathological grade (grade III versus grade I + II)	<0.001	0.031	5.291 (1.166–24.010)
Histological type (adenocarcinoma versus non-adenocarcinoma)	0.153		
Tumour stage (stage III + IV versus stage I + II)	0.811		
Tumour diameter	0.016	0.086	1.066 (0.912–1.247)
EGFR gene status (EGFR wild-type versus EGFR mutation)	0.010	0.069	0.332 (0.035–3.180)
70 keV CT value in AP	0.203		
70 keV CT value in VP	0.012	0.131	1.046 (0.944–1.158)
NIC in AP	0.003	0.371	0.653 (0.407–1.047)
NIC in VP	<0.001	0.765	1.042 (0.882–1.232)
λ_HU_ in AP	0.004	0.488	158.089 (0.677–36924.077)
λ_HU_ in VP	<0.001	0.001	0.020 (0.002–0.197)

keV, kilo electron volt; NIC, normalized iodine concentration; λ_HU_, the slope of the spectral curve. AP, arterial phase; VP, venous phase.

### The logistics regression analysis of the association of EGFR mutation status with the spectral CT parameters and clinic characteristics

Logistic regression analysis was performed on the association between EGFR mutation status and the spectral CT parameters and clinic characteristics. In the univariate regression analysis, smoking status, tumour histological type, Ki-67 expression level and the spectral CT quantitative parameters of the NIC in the AP and VP and λ_HU_ in the VP were significantly correlated with EGFR mutation status. Further multivariate regression analysis was used to correct for the interaction of various factors affecting EGFR mutation status. It was found that smoking status and the NIC in the VP were independently correlated with EGFR mutation status. Between the EGFR mutation group and the EGFR wild-type group, the OR of smoking status (comparing non-smoker with smoker) for EGFR mutation was 5.958 with a 95% confidence interval of 1.119–31.709, and the OR of the NIC in the VP (increased 1 Unit) for EGFR mutation was 1.102 with a 95% confidence interval of 1.040–1.168 (Table 6).

**Table 6:** Univariate and multivariate logistic regression analyses of EGFR mutation status.

	Univariate analysis	Multivariate analysis	
	P value	P value	OR (95% CI)
Sex (male versus female)	0.532		
Patient age	0.836		
Smoking status (non-smoker versus smoker)	0.019	0.036	5.958 (1.119–31.709)
Pathological grade (grade III versus grade I + II)	0.157		
Histological type (adenocarcinoma versus non-adenocarcinoma)	0.042	0.290	2.272 (0.496–10.410)
Tumour stage (stage III + IV versus stage I + II)	0.647		
Tumour diameter	0.200		
Ki-67 expression status (low level versus high level)	0.010	0.280	2.391 (0.491–11.642)
70 keV CT value in AP	0.987		
70 keV CT value in VP	0.785		
NIC in AP	0.041	0.954	0.996 (0.869–1.142)
NIC in VP	0.001	0.033	1.102 (1.040–1.168)
λ_HU_ in AP	0.053		
λ_HU_ in VP	0.007	0.364	0.397 (0.094–1.715)

keV, kilo electron volt; NIC, normalized iodine concentration; λ_HU_, the slope of the spectral curve. AP, arterial phase; VP, venous phase.

### Diagnostic implications

**Figure 3:**
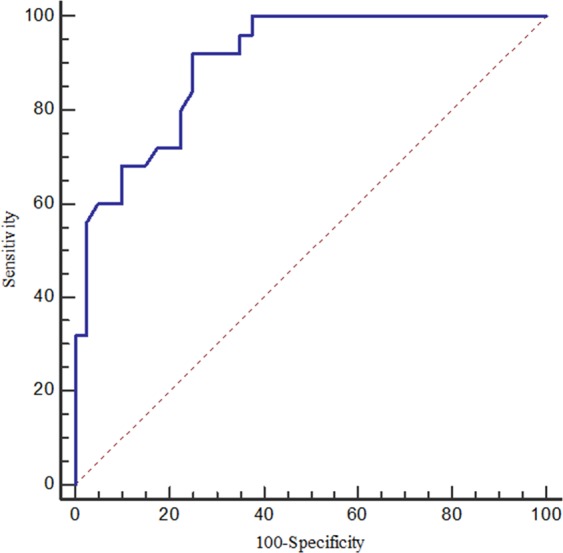
ROC curve of λ_HU_ in the venous phase for predicting Ki-67 expression in NSCLC.

**Figure 4:**
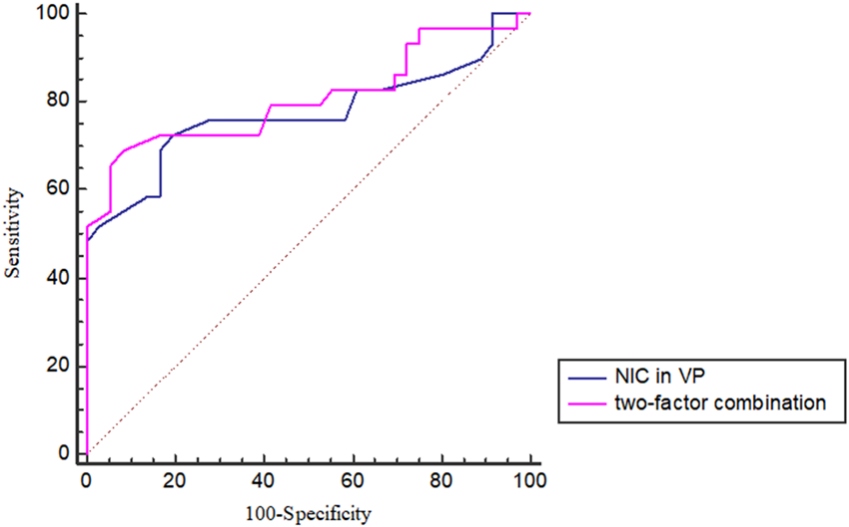
ROC curve for predicting EGFR gene mutation in NSCLC.

According to multivariate logistic regression analysis, the spectral CT quantitative parameter λ_HU_ in the VP was an independent factor influencing Ki-67 expression level, and the spectral CT quantitative parameter of the NIC in the VP was an independent factor influencing EGFR gene mutation. According to ROC curve analysis, the AUC of using λ_HU_ in the VP to predict the high Ki-67 expression of NSCLC was 0.901 with a sensitivity and specificity of 92% and 75%, respectively, and the diagnostic threshold was 2.16. The AUC of using the NIC in the VP to predict EGFR gene mutation in NSCLC was 0.772 with a sensitivity and specificity of 72.4% and 80.6%, respectively, and the diagnostic threshold was 0.41. Regarding the low sensitivity and specificity, two independent factors, smoking status and the NIC in the VP, were combined for ROC curve analysis. The AUC of the two-factor combination to predict EGFR gene mutation in NSCLC was 0.807 with a sensitivity and specificity of 72.4% and 85.8%, respectively (Figs. 3 and 4).

## Discussion

Ki-67 expression has long been regarded as a biological marker reflecting the aggressiveness of a tumour that can predict the prognosis of NSCLC patients. EGFR-TKIs have been reported as an effective treatment for NSCLC patients with EGFR mutations. Therefore, NSCLC patients, especially adenocarcinoma patients, are recommended to undergo EGFR mutation testing^20^. The results of the present study reveal that the 70 keV CT value in the VP and the NIC and λ_HU_ in both the AP and VP were significantly different between the high-level Ki-67 expression and low-level Ki-67 expression tumours. In addition, the NIC in the AP and VP and λ_HU_ in the VP had higher values in the EGFR-mutated tumours than in the EGFR wild-type tumours.

Enhanced CT values and iodine quantification in a lesion reflect the vascularity of the lesion. Additionally, the NIC serves as a benefit of spectral CT imaging in cancer characterization, and it was calculated to minimize the dose of contrast medium, the flow rate of injection, and individual differences in circulation in our study. We found a significant negative correlation among the 70 keV CT value in the VP, the NIC value in the AP and VP and Ki-67 expression—high expression of Ki-67 was usually correlated with a low 70 keV CT value in the VP and the NIC in the AP and VP. One possible explanation is that NSCLCs with radical proliferation may have an insufficient blood supply in terms of an increasing trend towards a large volume, which usually leads to intratumoural necrosis. The proliferative activity in a tumour is commonly evaluated based on the Ki-67 value, and we also found that the tumour diameter had a significant positive correlation with the Ki-67 value—the higher the expression of Ki-67 was, the faster the growth and the larger the tumour volume. Spira *et al*.^21^ reported that tumours with progressive growth appeared to have a large extent of tumour necrosis. The hypovascularity of a tumour before necrosis can affect tissue blood volume or vessel physiological function and can be reflected in the enhanced CT values and iodine concentration. In dual-energy spectral CT technology, the slope or change rate of the spectral curve has the ability to distinguish different materials because the different chemical compositions and organizations have different spectral curves^22,23^. Our study results showed that the λ_HU_ values for NSCLC in the AP and VP were higher in the low-level Ki-67 expression tumours than in the high-level Ki-67 expression tumours. These differences may be explained by the fact that a tumour with different Ki-67 expression values may have different compositions and organizations. Increased Ki-67 values are associated with the malignancy of tumours, and this kind of tumour is characterized by a complex internal structure, such as an abnormal nuclear/cytoplasmic ratio with cells, highly abundant macromolecular proteins, and compact extracellular space. Karaman *et al*.^24^ reported a strong negative correlation between the minimum apparent diffusion coefficient (ADC_min_) values in MRI and the Ki-67 proliferation index in NSCLC. Since the ADC value is always used to quantify the diffusion of water molecules in tissues, this result may suggest that a change in Ki-67 expression may influence the tumour internal structure. Furthermore, λ_HU_ in the VP was an independent factor influencing Ki-67 expression level; λ_HU_ in the VP reflects the difference in chemical compositions and organizations that are complex in fast proliferating tumours.

Regarding EGFR mutations, the NIC in the AP and VP and λ_HU_ in the VP were higher in tumours with EGFR mutations than in those without EGFR mutations, and the NIC in the VP was an independent factor influencing EGFR mutation. The clinical characteristic of smoking status was also an independent influencing factor. EGFR is a member of the type I tyrosine kinase receptor family, has intrinsic tyrosine-kinase activity and plays an important role in tumour development. EGFR can phosphorylate extracellular-regulated kinases (ERKs) and can impact cell proliferation to some extent^25^. The abnormal proliferation of tumour cells is one reason for tumour heterogeneity, and this situation resulted in different λ_HU_ values in the VP of the lesions between different EGFR mutation statuses. Previous studies have shown that the stimulation or inhibition of EGFR may cause differences in tumour-induced angiogenesis^26^, so the NIC, which represents the blood volume in the tumour, may be different according to the EGFR mutation status, results that are consistent with our study results. In tumour angiogenesis, vascular endothelial growth factor (VEGF) is one of the key stimulators of angiogenesis. Petit *et al*.^27^ has reported that EGFR might contribute to tumour angiogenesis partially through its effects on the expression of VEGF. Meng Li *et al*.^28^ explored the role of the NIC in the identification of EGFR mutation status in a cohort of East Asian patients with pulmonary adenocarcinoma. The NIC was an independent risk factor in their study, which is consistent with our results.

The ROC analysis was performed according to the results of the logistic regression. The ROC analysis in this study showed that the optimal diagnostic threshold of λ_HU_ in the VP for predicting the Ki-67 expression level was 2.16, resulting in a sensitivity of 92% and a specificity of 75%. This might be explained by the intratumoural structural changes correlated with different Ki-67 expression levels, which contribute to the difference in λ_HU_ in the VP. Nguyen *et al*.^29^ found that there was a significant difference between the maximum FDG uptake (SUVmax) and Ki-67 expression levels, and SUXmax was an independent factor influencing survival after tumour treatment. The results of research by Murakami *et al*.^30^ also showed a significant correlation between SUVmax and the Ki-67 expression index and tumour diameter. And Karaman *et al*.^24^ found that the ADCmin may be an useful parameter to predict the tumour Ki-67 expression index. However, their diagnostic efficacies for differentiating Ki-67 expression in NSCLC have not explicitly been described in their articles. The AUC of the NIC in the VP for predicting the presence of EGFR mutation was 0.772 in our study. Although this AUC value seems to be slightly low, it is still significantly higher than that of Meng Li *et al*.^28^, where they explored the role of the NIC for the identification of EGFR mutation status in a cohort of East Asian patients with pulmonary adenocarcinoma; the NIC, as an independent risk factor, had an AUC of 0.650 for the prediction of EGFR mutation in their research. Further, the combination of smoking status and the NIC in the VP promoted an AUC of 0.807 and a specificity of 85.8%.

There are certain limitations in the present study. First, this study focused on the application value of spectral CT, so all patients included should have previously undergone a spectral CT scan. The results were limited by the small number of cases and the retrospective design of the study, as selection bias could not be completely avoided; as is well known, EGFR mutation is usually more frequent in females than in males, but in the present study, the cohort did not show this association. Thus, further studies with larger sample sizes and a prospective design should be performed to validate the results of the present study. Second, the flow rate of injection was fixed at 3 ml/s and was not adapted for body weight. Although we normalized the IC of the tumour with that of the aorta, the results might still have been influenced by this scanning method, and the weight of the patient should be taken into consideration in the future. Third, we worked diligently to achieve a radiological-pathological match in sample analysis; however, a perfect correspondence between pathological samples and the ROI on the spectral CT images was difficult to achieve in practice. Fourth, only limited sections were measured in our study, and although an average value was calculated, measurement deviations could not be avoided completely. Fifth, we focused on NSCLCs with solid portions, and pure ground glass nodules were not analysed because of their poor expression of Ki-67.

## Conclusions

Tumour pathological grade and λ_HU_ in the VP were independent factors influencing the Ki-67 expression level, and smoking status and NIC in the VP were independent factors influencing EGFR mutation. Furthermore, λ_HU_ in the VP was useful for predicting the Ki-67 expression level, and the combination of smoking status and NIC in the VP was helpful for predicting EGFR mutation in NSCLC.

## Methods

### Patients

This retrospective study was approved by the Ethics Committee of Shanghai Ren Ji Hospital, the experimental protocols were performed in accordance with the approved guidelines, and the requirement for informed consent was waived because of the retrospective nature of the study. A retrospective analysis of data from patients who underwent dual-phase enhanced DESCT before surgery from March 2015 through October 2018 was performed. The inclusion criteria were (1) patients with solid types of non-small-cell lung cancer confirmed after surgery; (2) patients who underwent surgical resection within 2 weeks after CT examinations; and (3) patients with both Ki-67 and EGFR test results after surgery. The exclusion criteria were as follows: (1) patients who had undergone preoperative chemoradiotherapy, chemotherapy, radiotherapy or targeted therapy; (2) patients with metastatic tumours; and (3) patients with severe imaging artefacts. For patients with multifocal or multicentric lung cancer, only the largest surgical pathology-confirmed tumours were included. Ultimately, 65 lung cancer patients (43 men and 22 women; mean age, 64.29 years; range, 34–83 years) were included in the present study. All patients had dual-phase enhanced dual-energy spectral CT images and detailed pathological records of Ki-67 values and EGFR situations.

### Dual-energy spectral CT imaging

All patients were scanned in a supine position with the hands above the head on a 64-row single-source, dual-energy spectral CT Discovery CT750 HD scanner (GE Healthcare, Milwaukee, WI, USA). In our institution, dual-phase contrast-enhanced CT examination for lung cancer is performed routinely, and the radiation dose for each contrast-enhanced CT examination was approximately 8.40 mGy in the volumetric CT dose index (CTDIvol) for non-contrast CT scans and 7.37 mGy in the CTDIvol for each of the dual-phase contrast-enhanced CT scans. The dual-phase contrast-enhanced scans were performed with the dual-energy spectral imaging mode. Non-ionic iodinated contrast material (iopamidol, 370 mg iodine/ml; Shanghai Bracco Sine Pharmaceutical, Shanghai, China) was injected with a power injector at a dose of 1.35 ml/kg body weight at a rate of 3 ml/s through the hand vein. The dual-phasic enhancement CT for the arterial phase (AP) and venous phase (VP) started 35 and 90 seconds, respectively, after the administration of contrast agents. The dual-energy spectral CT mode scan parameters were as follows: instantaneous switch (<0.5 ms) between high (140 kVp) and low (80 kVp) tube voltages, a tube current of 375 mA, a tube rotation time of 0.6 seconds, a helical pitch of 1.375:1, a scan field of view of large body, and a collimation of 64 × 0.625 mm.

### Image analysis

All images were reconstructed at a section thickness of 1.25 mm, and measurements were performed on an Advanced Workstation (AW 4.6; GE Healthcare, USA) with Gemstone Spectral Imaging (GSI) Viewer software (GE Healthcare). Two experienced radiologists—both with 10 years of experience in chest imaging and who were blinded to the clinical and pathological information—analysed the images and measured data in consensus. Three consecutive image sections containing the section of the largest axial diameter of the lesion and the section above and below were chosen for measurement. Three regions of interest (ROIs) were drawn on the solid parts of the lesions of the three sections (areas close to 1/2 to 2/3 of the lesion area) while avoiding obvious necrosis, vessels, calcification and cystic portions. The 70 keV CT value was measured in the 70 keV monochromatic images. The iodine concentrations (ICs) of the lesions and aorta were measured from the iodine-based material decomposition images, and the CT values of lesions were measured from the 101 sets of virtual monochromatic images (with energy levels from 40 to 140 keV) by the GSI viewer software package. Then, average values were calculated from the measurements of the three consecutive image slices to minimize the bias of the measurement. The IC measurement itself was also influenced by various factors, such as the total dose of contrast medium, the flow rate of injection, and individual differences in circulation. To minimize the influence of variations in patients, the IC value of lung lesions was normalized to that of the aorta at the same section on the same iodine-based material decomposition image to calculate a normalized iodine concentration (NIC): ${\rm{NIC}}=I{C}_{{\rm{lesion}}}/I{C}_{{\rm{aorta}}}$. The range of 40 to 100 keV in the spectral attenuation was chosen to calculate the slope of the spectral CT curve. This range was chosen because the spectral attenuation curve between 100 and 140 keV was almost flat and because the slope in the 40–100 keV range was larger to improve the sensitivity of describing different slopes of spectral attenuation curves than the whole range. The slope of the spectral curve was calculated according to the formula:

$${\lambda }_{HU}=\frac{(CT\,valu{e}_{40keV}-CT\,valu{e}_{100keV})}{60}.$$

### Ki-67 expression and EGFR mutation analysis

The expression of Ki-67 in surgical specimens was analysed using a standard avidin-biotin immunohistochemistry (IHC) technique. The primary antibody for Ki-67 (Maixin Biotechnology Development Co., Ltd., Fuzhou, China, Kit-0005) was used according to the manufacturer’s instructions. The presence of EGFR mutations was determined by the amplification refractory mutation system (ARMS) method using a human EGFR gene mutation detection kit (AmoyDx, Xiamen, China, ADx-EG01), and the EGFR mutations investigated included exon 19 deletions, L858R, T790M, G719X, S768I, and L861Q. The procedures were performed according to the manufacturer’s instructions.

The evaluation and analysis were carried out by a trained pathologist who was blinded regarding the case data, and the Ki-67 value was calculated. The Ki-67 value was defined as the percentage of positive cancer cells, which was calculated as the percentage of Ki-67-positive tumour cells divided by the total number of tumour cells within one high-power field. Based on previous findings^10,31^, cut-off values of 20–30% distinguishing high versus low Ki-67 expression were used in many studies, and in our study, the median Ki-67 value was 30%, so we determined that if ≤30% of the nuclei were stained, the section was scored as having low expression of Ki-67; when >30% of the malignant nuclei were positive, the section was deemed to have high expression. The EGFR mutation positive values and test results were analysed according to the manufacturer’s instructions and the experience of the pathologist. Based on the outcome of the EGFR test, the tumours were divided into the EGFR mutation group and the EGFR wild-type group.

### Statistical analysis

All statistical calculations were performed with dedicated statistical software packages (SPSS, version 24.0, SPSS, Chicago, IL, USA; MedCalc, version 12.0, MedCalc Software, Mariakerke, Belgium). Quantitative variables were expressed as the means ± standard deviations (SDs). All reported P values were two-sided, and P < 0.05 was considered statistically significant. The two-sample t-test was used to assess the difference between spectral CT parameters for different Ki-67 expression levels or the EGFR mutation status. The logistics regression analyses were performed to identify independent factors in order to predict the Ki-67 expression levels and EGFR mutation status. Receiver operating characteristic (ROC) curves were generated to help establish the threshold values and the diagnostic capabilities for the significant spectral CT parameters for predicting Ki-67 expression and EGFR mutation. The optimal threshold value was evaluated by the Youden index. The diagnostic capability was determined by calculating the area under the ROC curve (AUC), and the sensitivity and specificity for each spectral CT parameter were obtained.

## Data Availability

The datasets generated and analysed during the current study are available from the corresponding authors upon reasonable request.

## References

[1] Siegel R, Ma J, Zou Z, Jemal A (2014). Cancer statistics, 2014. CA: a cancer journal for clinicians.

[2] Scholzen, T. & Gerdes, J. The Ki-67 protein: from the known and the unknown. *Journal of cellular physiology* **182**, 311–322, doi:10.1002/(sici)1097-4652(200003)182:3<311::aid-jcp1>3.0.co;2-9 (2000). 10.1002/(SICI)1097-4652(200003)182:3<311::AID-JCP1>3.0.CO;2-9 10653597

[3] Zolzer F, Speer A, Pelzer T, Streffer C (1995). Evidence for quiescent S- and G2-phase cells in human colorectal carcinomas: a flow cytometric study with the Ki-67 antibody. Cell proliferation.

[4] Li R (2004). Ki-67 staining index predicts distant metastasis and survival in locally advanced prostate cancer treated with radiotherapy: an analysis of patients in radiation therapy oncology group protocol 86-10. Clinical cancer research: an official journal of the American Association for Cancer Research.

[5] Tao M, Chen S, Zhang X, Zhou Q (2017). Ki-67 labeling index is a predictive marker for a pathological complete response to neoadjuvant chemotherapy in breast cancer: A meta-analysis. Medicine.

[6] Indinnimeo M (2000). Immunohistochemical assessment of Ki-67 as prognostic cellular proliferation marker in anal canal carcinoma. Journal of experimental & clinical cancer research: CR.

[7] Alexandrakis MG (2004). Ki-67 proliferation index: correlation with prognostic parameters and outcome in multiple myeloma. American journal of clinical oncology.

[8] Martin B (2004). Ki-67 expression and patients survival in lung cancer: systematic review of the literature with meta-analysis. British journal of cancer.

[9] Warth A (2014). Tumour cell proliferation (Ki-67) in non-small cell lung cancer: a critical reappraisal of its prognostic role. British journal of cancer.

[10] Hommura F (2000). Prognostic significance of p27KIP1 protein and ki-67 growth fraction in non-small cell lung cancers. Clinical cancer research: an official journal of the American Association for Cancer Research.

[11] Yamashita S (2011). Ki-67 labeling index is associated with recurrence after segmentectomy under video-assisted thoracoscopic surgery in stage I non-small cell lung cancer. Annals of thoracic and cardiovascular surgery: official journal of the Association of Thoracic and Cardiovascular Surgeons of Asia.

[12] Sofocleous CT (2013). Ki 67 is an independent predictive biomarker of cancer specific and local recurrence-free survival after lung tumor ablation. Annals of surgical oncology.

[13] Maemondo M (2010). Gefitinib or chemotherapy for non-small-cell lung cancer with mutated EGFR. The New England journal of medicine.

[14] Mitsudomi T (2010). Gefitinib versus cisplatin plus docetaxel in patients with non-small-cell lung cancer harbouring mutations of the epidermal growth factor receptor (WJTOG3405): an open label, randomised phase 3 trial. The Lancet. Oncology.

[15] Rosell R (2012). Erlotinib versus standard chemotherapy as first-line treatment for European patients with advanced EGFR mutation-positive non-small-cell lung cancer (EURTAC): a multicentre, open-label, randomised phase 3 trial. The Lancet. Oncology.

[16] Salto-Tellez M (2011). Clinical and testing protocols for the analysis of epidermal growth factor receptor mutations in East Asian patients with non-small cell lung cancer: a combined clinical-molecular pathological approach. Journal of thoracic oncology: official publication of the International Association for the Study of Lung Cancer.

[17] Zhang D, Li X, Liu B (2011). Objective characterization of GE discovery CT750 HD scanner: gemstone spectral imaging mode. Medical physics.

[18] Chae EJ (2008). Clinical utility of dual-energy CT in the evaluation of solitary pulmonary nodules: initial experience. Radiology.

[19] Hou WS (2015). Differentiation of lung cancers from inflammatory masses with dual-energy spectral CT imaging. Academic radiology.

[20] NB L (2014). Molecular testing for selection of patients with lung cancer for epidermal growth factor receptor and anaplastic lymphoma kinase tyrosine kinase inhibitors: American Society of Clinical Oncology endorsement of the College of American Pathologists/Internati. Journal of Clinical Oncology.

[21] Spira D (2013). Assessment of tumor vascularity in lung cancer using volume perfusion CT (VPCT) with histopathologic comparison: a further step toward an individualized tumor characterization. Journal of computer assisted tomography.

[22] Karcaaltincaba M, Aktas A (2011). Dual-energy CT revisited with multidetector CT: review of principles and clinical applications. Diagnostic and interventional radiology (Ankara, Turkey).

[23] De Cecco CN (2012). Dual-energy CT: oncologic applications. AJR. American journal of roentgenology.

[24] Karaman A (2015). Correlation of diffusion MRI with the Ki-67 index in non-small cell lung cancer. Radiology and oncology.

[25] Liebmann C (2001). Regulation of MAP kinase activity by peptide receptor signalling pathway: paradigms of multiplicity. Cellular Signalling.

[26] van Cruijsen H, Giaccone G, Hoekman K (2005). Epidermal growth factor receptor and angiogenesis: Opportunities for combined anticancer strategies. International journal of cancer.

[27] Yen LBN (2002). Differential regulation of tumor angiogenesis by distinct ErbB homo- and heterodimers. Molecular Biology of the Cell.

[28] Li Meng, Zhang Li, Tang Wei, Jin Yu-Jing, Qi Lin-Lin, Wu Ning (2018). Identification of epidermal growth factor receptor mutations in pulmonary adenocarcinoma using dual-energy spectral computed tomography. European Radiology.

[29] Nguyen XC (2007). FDG uptake, glucose transporter type 1, and Ki-67 expressions in non-small-cell lung cancer: correlations and prognostic values. European journal of radiology.

[30] Murakami S (2010). Correlation of 18F-fluorodeoxyglucose uptake on positron emission tomography with Ki-67 index and pathological invasive area in lung adenocarcinomas 30 mm or less in size. European journal of radiology.

[31] Jakobsen JN, Sorensen JB (2013). Clinical impact of Ki-67 labeling index in non-small cell lung cancer. Lung cancer (Amsterdam, Netherlands).

